# LAPAROENDOSCOPIC TRANSGASTRIC RESECTION OF SUBEPITHELIAL JUXTACARDIAC TUMORS

**DOI:** 10.1590/0102-6720201700020014

**Published:** 2017

**Authors:** Luiz Gustavo DE-QUADROS, Roberto Luiz KAISER-JUNIOR, Josemberg Marins CAMPOS, Valter Nilton FELIX, Mário FLAMINI-JÚNIOR, Maurício VECCHI, André Teixeira, Marcelo Falcão DE-SANTANA, Idiberto José ZOTARELLI-FILHO

**Affiliations:** 1 Kaiser Clinic and Hospital, Endoscopy, São José do Rio Preto, SP, Brazil;; 2 Hospital Beneficência Portuguesa de São José do Rio Preto, Endoscopy, São José do Rio Preto, SP, Brazil;; 3 School of Medicine of ABC, Santo André, SP, Brazil;; 4 Universidade Federal de Pernambuco, Recife, PE, Brazil;; 5 Orlando Health, Medicine, Orlando, Florida, USA;; 6 Faculty of Medicine, University of São Paulo, São Paulo, SP, Brazil.

**Keywords:** Videoassisted surgery, Laparoscopy, Endoscopy, Stomach neoplasms.

## Abstract

**Background:**

With a prevalence of 0.4-3.5%, subepithelial lesions of the upper digestive tract are discovered during endoscopic procedures. Treatment depends on etiological and pathophysiological information, ability to diagnose and the different technical resources available.

**Aim:**

To demonstrate the effectiveness of a surgical technique that combines endoscopy and videolaparoscopy in the transgastric resection of subepithelial juxtacardic lesions.

**Method:**

The patients were assisted with a technical combination between endoscopy and laparoscopy. After diagnosis of subepithelial tumor, intraoperative endoscopy was performed after pneumoperitoneum and placement of laparoscopic tweezers. Through endoscopy, the following steps were performed: demarcation of surgical margins, visualization of the intragastric image for the laparoscopic procedure and removal of the surgical specimen. By laparoscopy the following steps were performed: intragastric intra-abdominal access, resection of the part and closure of the gaps.

**Results:**

This technique was applied in two cases in order to evaluate its initial results. There were two videolaparoendoscopic resections of juxtacardiac gastric tumors of the posterior wall. Both had their endoscopic diagnosis confirmed. After laparoendoscopic and tomographic and/or ecoendoscopic diagnostic complementation and preoperative performance, the laparoendoscopic procedure was indicated. The patients had a good recovery, with a short hospitalization time and no complications.

**Conclusion:**

The combined use of videolaparoscopy and endoscopy is a safe and effective technique for transgastric resection of juxtacardiac subepithelial lesions. It may be important for definitive diagnosis of the tumor.

## RESUMO

**Racional:**

Lesões subepiteliais do trato digestivo superior são descobertas durante procedimentos endoscópicos com prevalência de 0,40-3,5%. Seu tratamento parte de conhecimento etiológico, fisiopatológico, capacidade diagnóstica e recursos técnicos variados.

**Objetivo:**

Demonstrar a eficácia de técnica cirúrgica que combina videolaparoscopia e endoscopia para ressecção transgástrica de lesões subepiteliais justacárdicas e seus resultados preliminares.

**Método:**

Os pacientes foram assistidos com uma combinação técnica entre endoscopia e laparoscopia. Após o diagnóstico de tumor sub-epitelial justacárdico endoscopia intra-operatória foi realizada após confecção do pneumoperitônio e colocação das pinças laparoscópicas. Através da endoscopia realizou-se os seguintes passos: demarcação de margens cirúrgicas, visualização da imagem intragastrica para o procedimento laparoscópico e retirada de peça cirúrgica; pela laparoscopia realizou-se os seguintes passos: acesso intragástrico por via intra-abdominal, ressecção da peça e fechamento das brechas.

**Resultados:**

Esta técnica foi aplicada em dois casos afim de serem avaliados seus resultados inicias. Foram duas ressecções videolaparoendoscópica de tumores gástricos justacárdicos, de parede posterior. Ambos tiveram seu diagnóstico endoscópico de tumores subepiteliais justacárdicos. Após complementação diagnóstica tomográfica e/ou ecoendoscópica e realização de pré operatório foi indicado o procedimento laparoendoscópico. Os pacientes tiveram recuperação muito satisfatória, com pequeno tempo de internação e sem complicações.

**Conclusão:**

O uso combinado de videolaparoscopia e endoscopia constitui técnica segura e eficaz para a ressecção transgástrica de lesões subepiteliais justacárdicas. Pode ser importante para diagnóstico definitivo da tumoração.

## INTRODUCTION

Subepithelial lesions are discovered during endoscopic procedures with a prevalence ranging from 0.4-3.5%^19^ ^,^ ^26^ and more common in the stomach, esophagus and duodenum^10^ ^,^ ^14^. In most cases they are present below the mucosa; sometimes, however, covered by inflamed or ulcerated mucosa^3^ ^,^ ^16^ ^,^ ^19^. Epidemiological data show that one in 300 routine endoscopies reveals a subepithelial lesion covered by normal-appearing mucosa^11^.

In most cases, biopsies do not provide definitive histological diagnosis because they do not reach the tumor or provide material that is too scarce for analysis^8^. The results of echoendoscopy, ultrasonography, computed tomography and magnetic resonance imaging are almost always not precise in the characterization of subepithelial lesions, especially in the smaller ones than 2.0 cm^12^ ^,^ ^24^.

They are almost always benign, especially lipomas, ectopic pancreas and leiomyomas^1^ ^,^ ^17^. The risk that the tumor is malignant always exists^27^, but in general, with no propensity to invade adjacent structures. This is the reason for the possibility of limited surgical resections, facilitated by the use of minimally invasive techniques, which may be associated with endoscopy^14^ ^,^ ^27^. In this context, laparoscopic surgery has become the standard for various surgical procedures of the stomach, with concomitant diagnostic and therapeutic intent^18^ ^,^ ^21^.

According to the National Comprehensive Cancer Network (NCCN), a laparoscopic approach is recommended for resection of subepithelial gastrointestinal tumors smaller than 5 cm^23^. However, for lesions located in regions of difficult access to gastric areas, particularly those close to the gastroesophageal and posterior wall junction, new clinical trials have sought to evaluate the efficacy of a combination of endoscopic and laparoscopic or robotic techniques^14^ ^,^ ^26^. In this sense, De Matteo et al.^5^ recommended surgical margins of 1-2 cm, demarcated by endoscopy, to provide safety for laparoscopic resection of the lesions.

Another relevant aspect is that endoscopic resections have always been widely used to remove tumors located in the mucosa or submucosa^7^ ^,^ ^13^, but present a significant risk of perforation if the muscular layer is involved by the tumor^7^ ^,^ ^26^, prescribing greater safety to the combination of endoscopic techniques and videolaparoscopic^20^, both for enucleations and for segmental resection of the gastric wall in the surgical approach of subepithelial lesions.

The purpose of this study was to present the technique proposed by the authors for laparoendoscopic resection of the gastric subepithelial tumors and to demonstrate its efficacy and results in initial experience.

## METHOD

### Technique

By endoscopy (Olympus CV 160^®^) a circular area around the lesion is demarcated by cauterization using a hook knife, giving a margin of safety of about 1-2 cm. Meanwhile, the surgical team initiates laparoscopy by installing two 5 mm subcostal trocars and one 10-11 mm supraumbilical, respectively, for access the Ultracision^®^ (Ethicon) harmonic scalpel, forceps and 30° videocamera into cavity. The scalpel and the forceps are introduced into the stomach, already inflated by the endoscope, by two orifices of about 5 mm in the anterior wall of the organ, about 10 cm from the cardia. We then proceed to the resection of the demarcated area, guided by an endoscopic image. The gastric segment containing the lesion is removed by the mouth, using the Olympus^®^ tripod, minimizing cavitary contamination. The laparoscopic time closes the resection site and the gastric lumen access holes, with continuous sutures in total plane with Ethibond^®^ 3.0. At the end, the suture is tested by means of the “maneuver of the tire repairman”. The technical steps of the procedure are seen in Figures 2 and 3.

**FIGURE 1:**
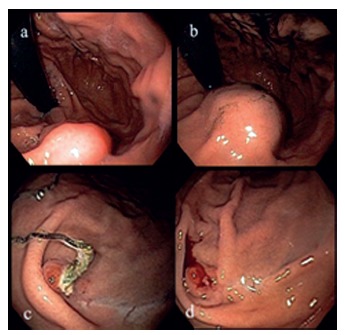
Diagnostic aspects of juxtacardiac tumor: A and B) endoscopic aspect where posterior subepithelial lesion is observed in the posterior wall of the gastric body; C and D) endoscopic control images six months after resection (case 1)

**FIGURE 2:**
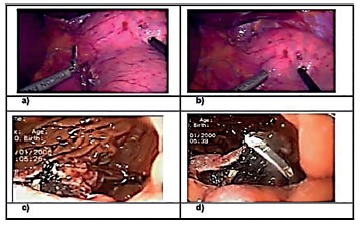
Images of the first case: A) introduction of forceps (right hand of the surgeon) and second transgastric access; B) transgastric access made with surgical materials (grasper and hemostatic scissors); C) endoscopic view and presentation of the lesion with apprehension forceps; D) resection of the lesion using Ultracision® (Ethicon)

**FIGURE 3:**
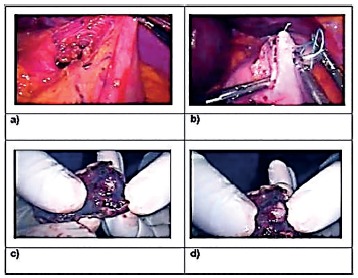
Images of the first case: A) local appearance after resection of the lesion; B) raffia at the site of resection of the lesion with Ethibond®2-0 (Ethicon); C) and D) specimen after oral endoscopic removal showing safety margins and tumor

## RESULTS

### This procedure was performed in two patients with these tumors.

The first referred to a 34-year-old woman who had used lansoprazole for a long time to control epigastric pain, aggravated in the last three months. Upper gastrointestinal endoscopy showed a elevated lesion approximately 1 cm in diameter, 2 cm below the esophagogastric transition, at the midpoint of the posterior wall, covered by normal-appearing mucosa with fibroelastic consistency. Computed tomography of the upper abdomen revealed a 1.4x1 cm nodular lesion in the posterior wall of the stomach, with no other changes worthy of note, whereas echoendoscopy suggested a leiomyoma of about 1 cm, but did not obtained biopsy material sufficient for definitive diagnosis (Figure 1). It was chosen as treatment by resection of the lesion using the technique described combining endoscopy and videolaparoscopy. The anatomopathological examination confirmed the diagnostic hypothesis of leiomyoma, attesting to free surgical margins. There was no complication and the patient received a liquid diet and hospital discharge on the first postoperative day. Endoscopic follow-up was performed four months after the procedure, visualizing a juxtacardiac scar of about 3 cm of the posterior wall. She was asymptomatic at the time.

**FIGURE 4:**
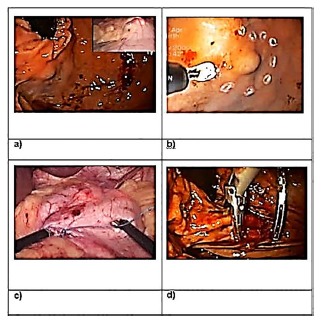
Images of the second case: A) visualization and endoscopic location in retrovision; B) endoscopic margin marking; C) laparoscopic intragastric access; D) intragastric resection under vision and endoscopic control

**FIGURE 5:**
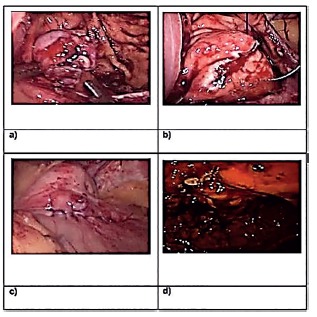
Images of the second case: A) capture of intragastric lesion for “delivery to endoscope”; B) gap raffia at resection site with 2-0 PDS thread; C) final intra-abdominal appearance after closure; D) final intragastric appearance.

**FIGURE 6:**
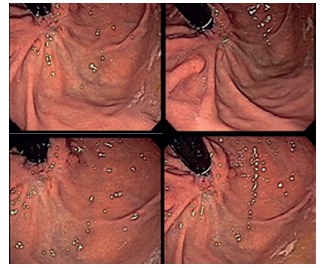
Control endoscopy images after resection: A and B) at three months; C and D) with six months

The second case referred to a 41-year-old man in clinical treatment of chronic gastritis. In control endoscopy trol was found gastric subepithelial lesion, posterior wall measuring approximately 7x5 mm. On endoscopic ultrasonography the lesion was hypoechoic, homogeneous, measuring 9.8x5.8 mm, and originated in the muscular layer. A stromal tumor was hypothesized, and it was decided resection using the combined technique of videolaparoscopy and endoscopy, due the patient’s wish was to get rid of any type of tumor that might exist. The technical procedure was the same as described in the method. When the pneumoperitoneum was established, a high digestive endoscopy was performed, with visualization of the lesion in the posterior wall, adjacent to the cardia. It was demarcated, with cautery, obeying the safety margin of 2 cm. Two 5 mm incisions were then made in the anterior wall of the stomach, already inflated by the endoscope, about 10 cm from the cardia, to forceps and harmonic scalpel, used to initiate the resection of the previously demarcated area, guided by endoscopic. The remainder procedure proceeded as previously described (Figure 4). This case presented a certain technical difficulty, and it was then decided to join the two holes of the anterior gastric wall, forming an opening of about 4 cm in extension, with wide access to the gastric lumen. The image acquisition was transferred to the videocamera and the resection of the marked segment of the posterior gastric wall was completed. The surgical specimen was removed orally. The resection region and the anterior wall opening of the stomach were closed with continuous sutures, total PDS^®^ 3-0 plane. The technical steps of the procedure can be seen in Figures 5 and 6

For safety, the release of liquid diet was delayed for the second postoperative day and hospital discharge for the third. There were no clinical or surgical complications. The anatomopathological and immunohistochemical examinations of the surgical specimen revealed GIST.

With the patient asymptomatic, endoscopic follow-up was performed three months after the procedure, showing only scars of about 4 cm of anterior and posterior juxtacardicac walls.

## DISCUSSION

There is a lack of randomized multicenter studies investigating resection of subepithelial tumors by laparoscopy combined with endoscopy. The present paper thus contributes to the literature, describing the success of the videolaparoendoscopic approach, a technique that provides accurate localization and safe resection of small tumors. This method uses a minimally invasive approach to tumor resection.

In this context, leiomyomas are benign lesions, most often located in the distal esophagus. Surgical procedure is taken as mandatory when they are larger than 4 cm, and when there are symptoms^9^. In most cases, however, the lesions are small and the asymptomatic patients are monitored by endoscopy^13^, even for lack of a better proposition of a minimally invasive procedure that can define concomitant diagnosis and treatment and solve the problem at once, as happened in case 1.

Immunohistochemical markers such as CD34 and CD117 are important for the differentiation of subepithelial lesions such as GIST and leiomyoma, but the application of markers is only possible when an adequate amount of analysis material is available^2^ ^,^ ^6^, as occurred in case 2, in which the diagnosis was confirmed only after complete resection of the tumor.

Endoscopy is able to provide valuable information for the diagnosis of subepithelial lesions, including the size, mucosal aspect of the lesion, tumor consistency, and other signs that may predict the cause, such as the “cushion sign” in the lipomas and central prominence in the ectopic pancreas^14^ ^,^ ^27^. However, in most cases, the characterization of the endoscopic lesion is not definitive, even when applying “biopsies in the biopsy” and macrobiopsies, with the other examinations of the image not being enough^17^.

When they are restricted to the submucosa they can be endoscopically enucleated, with a success rate that may reach 92.3%^28^. But, a little deeper, carry considerable risk of perforation. This justifies the current effort to combine techniques to take them out safely, early, even small, avoiding costly and tense long-term monitoring.

In a retrospective study, the medical records of 1684 patients with subepithelial tumors of the upper gastrointestinal tract, detected during routine endoscopic examinations between 2004 and 2013, were analyzed. The mean tumor size was 8.7 mm. Mean follow-up of 47.3 months, with serial endoscopies, showed that the lesion size remained unchanged in 920 individuals (96.4%), but in 34 (3.6%) they increased in diameter by at least 25%^26^. This can be seen in two ways. The former considers regular endoscopy sufficient to monitor small subepithelial tumors. The second one calls for something that resolves such cases at once, avoiding weariness of the patient and the care team.

Studies have suggested that videolaparoscopy is a method capable of providing a curative approach for almost all gastric subepithelial lesions^25^ ^,^ ^26^. Moreover, this technique is considered safe, offering small morbidity and short hospitalization^29^. These facts could be proven in the presentation of the two cases described, stimulating the continuation of the experience that may lead to the new north in the conduction of the small subepithelial lesions of the stomach.

The greatest technical difficulty encountered in this study was the small field to work in. However, this technique allowed the resection of tumors without the need for large resections, maintaining the stomach with its preserved capacity and without the need for anastomoses.

## CONCLUSION

The combined use of videolaparoscopy and endoscopy was shown to be safe and effective in the resection of posterior wall subepithelial lesions. It may be important for definitive diagnosis of the tumor.
